# Institutional Questions

**Published:** 1899-08-26

**Authors:** 


### INSTITUTIONAL QUESTIONS.

**Paying Patients.**

"Hospital Secretary" writes: We have opened a pay side to this infirmary. Can you direct me to, say, half a dozen institutions of similar size to this where they receive paying patients that I may learn their tariff with a view to the preparation of one for this institution ?

Unfortunately, we are unable to give a direct answer to this question, for up to the present, although paying patients are received at many hospitals, a "pay side" is practically a novelty except in regard to St. Thomas's Hospital, where one of the blocks is used for paying patients under the name of St. Thomas's Home and is under the management of a resident medical officer who will either take entire charge of the cases or will meet in consultation such medical men as may be employed by the patients. At many cottage hospitals paying patients are received, and in this regard the following list of fees asked may be useful: Axminster Cottage Hospital (6 beds), paying patients not less than 15s. per week; Blandford Cottage Hospital (12 beds), private wards £1 11s. 6d. to £5 5s. per week; Bodmin, East Cornwall Hospital (15 beds), paying patients 12s. per week; Brackley Cottage Hospital (6 beds), paying patients 21s. and upwards weekly; Chislehurst, &c., Cottage Hospital (14 beds), paying patients one to two guineas per week; Droitwich, St.

John's Brine Baths Hospital for Poor Patients (32 beds), paying patients £1 2s. per week; Guernsey, Victoria Cottage Hospital (13 beds), paying patients two and a half to three guineas per week; Manchester, Eccles and Patricroft Hospital (12 beds), paying patients from £1 1s. per week; Mirfield Memorial Hospital (18 beds), paying patients £1 1s. per week; Sherborne, Yeatman Hospital (24 beds), paying patients, £1 1s. per week. It must be remembered, however, that at most cottage hospitals, not only are special fees taken from those who might properly be called special cases, as in the above instances, but it is a general rule for all patients to pay something, say, half-a-crown a week and upwards according to their means. What seems to us a fair and proper arrangement would be for all people admitted to hospitals to pay according to their means. The very poor would pay nothing, but all who are able to contribute at all should contribute something, the amount varying according to the patient's means, it being always understood that this contribution in no way alters the fact that the benefit of hospital treatment is a charity. So long as the amount of the weekly contribution is less than the weekly average cost of the patient, clearly the patient is receiving a charitable gift, and of this charity the medical officer will give his share in the way of professional attendance. But directly the contribution becomes equal to the expense then the excess should be equally divided between the institution and the medical man. It would be unreasonable, we think, to expect a medical man to give his attendance for nothing to people to whom the hospital subscribers were making no gift. With an arrangement such as this there would be no hard and fast line between the paying and the non-paying patient. The "pay side," however, might properly be reserved for those who pay as much as or more than they cost.

